# Combination with Red ginseng and *Polygoni Multiflori* ameliorates highfructose diet induced metabolic syndrome

**DOI:** 10.1186/s12906-016-1063-7

**Published:** 2016-03-09

**Authors:** Min Chul Kho, Yun Jung Lee, Ji Hun Park, Jeong Dan Cha, Kyung Min Choi, Dae Gill Kang, Ho Sub Lee

**Affiliations:** College of Oriental Medicine and Professional Graduate School of Oriental Medicine, Wonkwang University, Shinyong-dong, Iksan, Jeonbuk, 570-749 Korea; Hanbang Body-fluid Research Center, Wonkwang University, Shinyong-dong, Iksan, Jeonbuk, 570-749 Korea; Brain Korea (BK)21 plus team, Professional Graduate School of Oriental Medicine, Wonkwang University, Iksan, Jeonbuk, 540-749 Republic of Korea; Department of Research Development, Institute of Jinan Red Ginseng, Jinan, Korea

**Keywords:** Red ginseng, *Polygoni Multiflori* Radix, Metabolic syndrome, Vascular inflammation, Stetohepatitis

## Abstract

**Background:**

Metabolic syndrome such as dyslipidemia, hypertension, obesity, impaired glucose tolerance and fatty liver, can be caused by modification of diet by means of overconsumption of high fructose diet. This study was designed to investigate whether combination with Red ginseng and *Polygoni Multiflori* Radix (RGPM), widely used traditional herbal medicine, ameliorates on highfructose (HF) diet-induced metabolic syndrome.

**Methods:**

SD rats were fed the 60 % HF diet with/without rosiglitazone, and RGPM 100, 300 mg/kg/day, respectively. All groups received regular diet or HF diet, respectively, for 8 weeks. The last three groups treatment of rosiglitazone and RPGM orally for a period of 6 weeks.

**Results:**

Chronic treatment with RGPM significantly decreased body weight, fat weight and adipocyte size. RGPM significantly prevented the development of the metabolic disturbances such as hypertension, hyperlipidemia and impaired glucose tolerance. RGPM also led to increase in high density lipoprotein level in the HF group. RGPM suppressed high-fructose diet induced vascular inflammation marker expression such as adhesion molecules and ET-1 in aorta as well as increasing of C-reactive protein (CRP) levels in plasma. Similarly, RGPM attenuated hepatic lipid accumulation by inhibition of monocyte chemoattractant protein-1 (MCP-1) expression.

**Conclusion:**

An administration of RGPM may be a beneficial therapy for the treatment of metabolic syndrome through the improvement of hypertension, obesity, hyperlipidemia, vascular inflammation and insulin resistance.

## Background

Metabolic syndrome which is caused by disorder of signal delivery system of insulin hormone, such as insulin resistance and glucose metabolism in muscles, tissues of fat and liver. There are numerous people who have insulin resistance before diabetes disease except type 2 diabetes. Also, it is well known as an important factor of cardiovascular disease and stroke [1–3]. A damage of vascular endothelial cell is known to be a decisive factor of conditional pathophysiology of cardiovascular disease as arteriosclerosis which is caused by secretion of cell adhesion molecules, endothelin-1 (ET-1) and inflammatory cytokine [4]. Especially, it is reported that vascular inflammation which caused a disease with ischemia and necrosis of tissues, also it has various symptoms by invasion of all vascular not be related by a size, a location and a type [5, 6]. In metabolic syndrome, the liver is profoundly affected by excess dietary nutrients from the intestines and inflammatory adipocytokines from enlarged visceral adipose tissues. Thus, fatty liver is considered as a representative of metabolic syndrome [7–9]. Nonalcoholic fatty liver disease (NFLAD) induces a broad spectrum of manifestation of fatty liver, ranging from steatosis alone, steatosis with inflammation (stetohepatitis), steatosis with hepatocyte injury, or steatosis with sinusoidal fibrosis in relation to the progress of the pathological state [10]. Recently, natural substances and materials based on traditional medicines are interested for the prevented or obstructed diseases of fatty liver, hypertension, high cholesterol and diabetes. Thus, the demand has increased that an interest of treatment effects has risen [11–13].

Red ginseng (RG), is produced by steaming and drying fresh and raw ginseng. During the steaming process, ginsenosides produce chemical changes that confer the potential to induce special physical activities [14]. Several studies already reported that RG exhibits anti-inflammation, anti-oxidant, anti-diabetes and anti-aging effects [15–18]. *Polygoni Multiflori* Radix (PM), is originated from the root of Polygonum multiflorum Thunb, have many biological activities such as anti-oxidant, anti-tumor, anti-aging and regulate lipid metabolism [19, 20]. Those two herbs have been used for more than 2000 years in Asia as a traditional medicine and dietary supplement for health [21]. However, the effect combination with Red ginseng and *Polygoni Multiflori* Radix on high fructose (HF) diet animal model has not been yet reported. Thus, the present study was designed to determine whether combination with Red ginseng and *Polygoni Multiflori* Radix (RGPM) improves high fructose diet-induced metabolic syndrome.

## Methods

### Preparation of Red ginseng and *Polygoni Multiflori* Radix

The Red ginsengextract was purchased from the Institute of Jinan Red ginseng, is a commercially available product (Jinan, Jeonbuk Province, Korea), and *Polygoni Multiflori* Radix was purchased from Herbal Medicine Co-operative Association, Mujin, Jeonbuk Province, Korea, respectively. A voucher specimen (No. HBI141) was deposited in the herbarium of the Professional Graduate School of Oriental Medicine, Wonkwang University, Iksan, Jeonbuk, South Korea. The dried *Polygoni Multiflori Radix* (400 g) was boiled with 4 L of distilled water at 100 °C for 2 h. The extract was filtered through Whatman No.3 filter paper (Whatman International Ltd, England) and centrifuged at 990 × g for 20 min at 4 °C. Supernatant was concentrated using a rotary evaporator and then the resulting extract (19.929 g) was lyophilized by using a freeze-drier and retained at −70 °C until required. The combination of Red ginseng and *Polygoni Multiflori* Radix was mixed at a ratio of 1:1.

### Animal experiments and diet

All experimental procedures were carried out in accordance with the National Institute of Health Guide for the Care and Use of Laboratory Animals and were approved by the Institutional Animal Care and Utilization Committee for Medical Science of Wonkwang University. Seven week old male Sprague–Dawley (SD) rats were obtained from Samtako (Osan, Republic of Korea). All Rats were housed in a room with an automatically maintained at a temperature (23 ± 2 °C), humidity (50 ~ 60 %) and 12-h light/dark cycle throughout the experiments. After 1 week of acclimatization, animals were randomly divided into fivegroups (*n* = 10 per group), namely, (1) the control group (SD rats + regular diet + distilled water), (2) High-fructose control group (SD rats + 60 % HF diet + distilled water), (3) positive control group (SD rat + 60 % HF diet + rosiglitazone 10 mg/kg/day), (4) combination with Red ginseng and *Polygoni Multiflori* Radixgroup 1 (SD rats + 60 % HF diet + RPGM 1 100 mg/kg/day), (5) combination with Red ginseng and *Polygoni Multiflori* Radixgroup 2 (SD rats + 60 % HF diet + RPGM 2 300 mg/kg/day). The 60 % high fructose diet (HF) was purchased from Research Diet, Inc. USA. All groups received regular diet or HF diet, respectively, for 8 weeks. The last three groups treatment of rosiglitazone and RPGM orally for a period of 6 weeks. The regular diet was composed of 50 % starch, 21 % protein, 4 % fat and standard vitamins and mineral mix. The high fructose diet was composed of 60 % fructose, 20 % protein, 10 % fat and standard vitamins and mineral mix.

### Blood and tissue sampling

At the end of the experiments, the aorta, liver and adipose tissue (epididymal fat pads) were separated, frozen until analysis after rinsed with cold saline. The plasma was obtained from the coagulated blood by centrifugation at 3000 rpm 15 min at 4 °C. The separation of plasma was frozen at −80 °C until analysis.

### Measurements of blood pressure

Systolic blood pressure (SBP) was determined by using non-invasive tail-cuff plethysmogrphy method and recorded with an automatic sphygmotonography (MK2000; Muromachi Kikai, Tokyo, Japan). SBP was measured at −1, 2, 5, and 8 week, respectively. At least seven determinations were made in every session. Values were presented as the mean ± SEM of four measurements.

### Biochemical analysis of plasma lipids

The total cholesterol (T-Cho), high-density lipoprotein- (HDL-) cholesterol, low-density lipoprotein- (LDL-) cholesterol, triglyceride (TG), glutamic-oxaloacetic transaminase (GOT) andglutamic-pyruvic transaminase (GPT) levels in plasma were enzymatically measured using commercially available kits (ARKRAY, Inc., Minami-ku, Kyoto, Japan). The creactive protein (CRP) and leptin levels were measured based on ELISA method using commercial rat creative protein and leptin ELISA kit (Leptin Rat ELISA ab100773, abcam).

### Estimation of blood glucose and oral glucose tolerance test

The concentration of glucose in blood was measured which obtained from tail vein using glucometer (Onetouch® Ultra™) and Test Strip (Life Scan Inc., CA, USA), respectively.

The oral glucose tolerance test (OGTT) was performed 2 days apart at 7 weeks. For the OGTT, briefly, basal blood glucose concentrations were measured after 10 ~ 12 h of overnight food privation, then the glucose solution (2 g/kg body weight) was immediately administered via oral gavage, and fourth more tail vein blood samples were taken at 30, 60, 90 and 120 min after glucose administration.

### Protein preparation and Western blot analysis in the rat aorta and liver

Thoracic aorta and liver were homogenized in a buffer consisting of 250 mM sucrose, 1 mM EDTA, 0.1 mM phenylmethylsulfonyl fluoride, and 20 mM potassium phosphate buffer (pH 7.6). Large tissue debris and nuclear fragments were removed by two successive low-speed spins (3500 rpm, 5 min; 8000 rpm, 10 min, 4 °C). The recovered protein (40 *μ*g) was separated by 10 % SDS-PAGE and transferred electrophoretically to nitrocellulose membranes using a Mini-Protean II apparatus (Bio-Rad, Hercules, CA). Membranes were blocked with 5 % BSA powder in 0.05 % Tween 20-Tris-bufferd saline (TBS-T) for 1 h prior to incubation in the presence of primary antibodies to E-selectin, VCAM-1, ICAM-1, PPAR-*γ*, and *β*-actin (in aorta) and MCP-1 (in liver) (Santa Cruz Biotechnology, Santa Cruz, CA) at a final dilution of 1:1000 overnight at 4 °C. The blot was washed several times with TBS-T and incubated with the appropriate horseradish peroxidase-conjugated secondary antibody for 1 h. After the membrane was washed several times with TBS-T, incubated with horseradish peroxidase-conjugated secondary antibody for 1 h, and then the immunoreactive bands were visualized by using enhanced chemiluminescence (Amersham, Buchinghamshire, UK). The bands were analyzed densitometrically by using a Chemi-doc image analyzer (Bio-Rad, Hercules, CA, USA).

### Histopathological staining ofepididymal fat

Epididymal fat and liver tissues were fixed by immersion in 4 % paraformaldehyde for 48 h at 4 °C, and then incubated with 30 % sucrose for 2 days. Each fat and liver was embedded in OCT compound (Tissue-Tek, Sakura Finetek, Torrance, CA, USA), frozen in liquid nitrogen, and stored at −80 °C. Frozen sectionswere cut with a Shandon Cryotome SME (Thermo Electron Corporation, Pittsburg, PA, USA) and placed on poly-L-lysine-coated slide. Epididymal fat sections were stained with H&E. For quantitative histopathological comparisons, each sections were determined by Axiovision 4 Imaging/Archiving software (Axiovision 4, Carl Zeiss, Germany).

Liver sections were assessed by using Oil red O staining. Each sections were stained with Oil red O for 20 min at room temperature after rinsing with 60 % isopropyl alcohol and distilled water. Images of Oil red O stained liver were taken with a Axiovision 4 Imaging/Archiving software. For quantitative analysis, the average score of 10 ~ 20 randomly selected area was calculated by using NIH Image analysis software, Image J (NIH, Bethesda, MD, USA).

### Immunihistochemical staining of aortic tissues

Aorta tissues were fixed by immersion in 4 % paraformaldehyde for 48 h at 4 °C, and then incubated with 30 % sucrose for 2 days. Each fat and liver was embedded in OCT compound (Tissue-Tek, Sakura Finetek, Torrance, CA, USA), frozen in liquid nitrogen, and stored at −80 °C. Frozen sections for immunohistochemical staining were placed on poly-L-lysine-coated slide (Fisher scientific, Pittsburgh, PA, USA). Slides were immunestained by Invitrogen’s HISOTO-STAIN®-SP kits using the Labeled-[strept] Avidin-Biotin (LAB-SA) method. After antigen retrieval, slide were immersed in 3 % hydrogen peroxide for 10 min at room temperature to block endogenous peroxidase activity, and rinsed with PBS. After rinsed, slides were incubated with 10 % non-immune goat serum for 10 min at room temperature, and incubated with a primary antibodies of ET-1, VCAM-1 and E-selectin (1:200; Santa Cruz, CA, USA) in humidified chambers overnight at 4 °C. All slides were then incubated with biotinylated secondary antibody for 20 min at room temperature, and then incubated with horseradish peroxidase-conjugated streptavidin for 20 min at room temperature. Peroxidase activity was visualized by 3-amino-9-ethylcarbazole (AEC; Novex®, CA) substrate-chromogen system, and counterstaining with hematoxylin (Zymed, CA, USA). Images of aorta tissues were taken with aAxiovision 4 Imaging/Archiving software. For quantitative analysis, the average score of 10 ~ 20 randomly selected area was calculated by using NIH Image analysis software, Image J (NIH, Bethesda, MD, USA).

### Statistical analysis

All the experiments were repeated at least three times. The results were expressed as a mean ± S.E. The data was analyzed using SIGMAPLOT 10.0 program. The Student’s *t*-test was performed to determine any significant differences. *P* < 0.05 was considered as statistically significant.

## Results

### Effects of RGPM on changes in body weight and epididymal fat pads weight

At time of sacrifice, mean body weight was shown as in Table 1, HF-diet rats showed significantly increased body weight compared to control group. However, treatment of RGPM groups showed significant decrease in body weight compared to HF group. In addition, the overall weight gain in HF group was significantly increased compared to control group. However, treatment of RGPM groups showed significant decrease in gain weight compared to HF group.

**Table 1:** Effect of RGPM on body weight and epididymal fat pads

Groups	Cont.	HF	HF		
			Ros.	RGPM 1	RGPM 2
Initial BW (g)	234.4 ± 1.2	236.9 ± 1.4	233.4 ± 2.1	234.4 ± 2.1	235.9 ± 2.1
Terminal BW (g)	418.0 ± 8.5	461.1 ± 10.6**	430.9 ± 6.0^#^	427.3 ± 8.0^#^	426.8 ± 9.2^#^
Gain BW (g)	183.7 ± 9.6	229.3 ± 11.7**	203.0 ± 5.1^#^	193.1 ± 5.8^#^	190.0 ± 8.0^#^
Epididymal fat pads weight (g)	6.1 ± 0.6	11.3 ± 0.7**	8.4 ± 0.9^#^	8.7 ± 0.7^#^	8.5 ± 0.9^#^

Values were expressed as mean ± S.E. (*n* = 10). **p < 0.01 vs. Cont.; ^#^ *p* < 0.05 vs HF. *Abbreviations*: *HF* high fructose, *HF + Ros.* high fructose diet with rosiglitazone, *HF + RGPM* high fructose diet with Red ginseng and *Polygoni Multiflori* Radix, *BW* body weight

Moreover, HF diet results in a significant increase in epididymal fat pads weight. The weight of epididymal fat pads was 84.57 % higher than that of the HF diet group compared to control group. However, treatment of RGPM groups significantly reduced the epididymal fat pads weight (−46.73, −46.53 %) compared to HF diet group, respectively. Similarly, treatment of rosiglitazone was showed similar results with RGPM groups.

### Effect of RGPM on the morphology ofepididymal fat pads

**Fig. 1:**
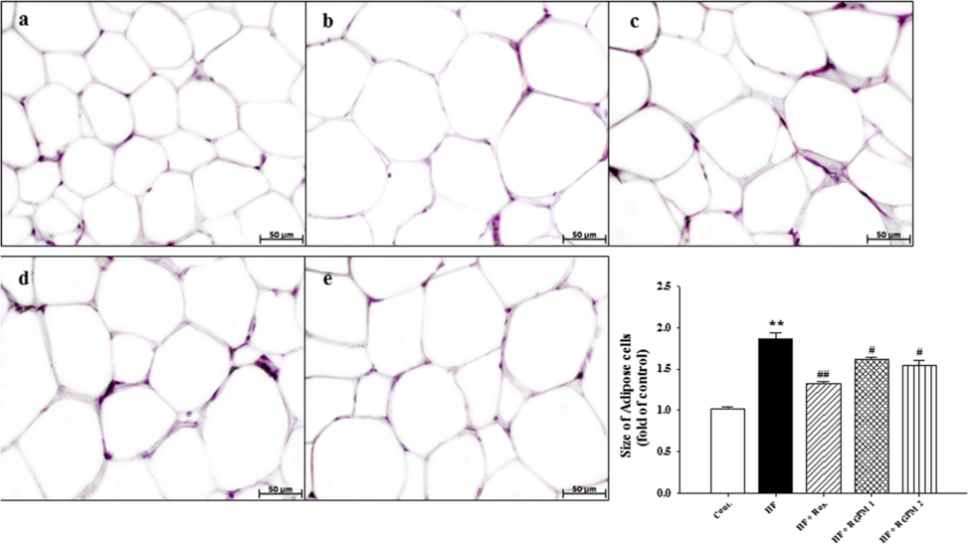
Effects of RGPM on adipocytes in HF diet rats. Representative microscopic photographs of H&E stained section of epididymal fat pads in HF diet rats. *Lower panel* indicated the size of adipose cells (magnification × 400). Values were expressed as mean ± S.E. (*n* = 4). **p < 0.01 vs Cont.; ^#^ *p* < 0.05, ^##^ *p* < 0.01 vs HF. *Abbreviations*: *HF* high fructose, *HF + Ros.* high fructose diet with rosiglitazone, *HF + RGPM* high fructose diet with Red ginseng and *PolygoniMultiflori* Radix. **a** control; **b** HF; **c** HF+Ros.; **d** HF+RGPM21; **e** HF+RGPM 2

Because RGPM effectively reduced the epididymal fat pads weight, we prepared frozen section of epididymal fat pads and stained with H&E. Histological findings as shown in Fig. 1 revealed hypertrophy of adipocytes in HF group compared to control group (+84.70 %, *P* < 0.01). However, treatment of RGPM groups showed significantly decreased the hypertrophy of adipocytes (−24.87 and −31.14 %, respectively, *P* < 0.05) (Fig. 1) compared to HF group. Similarly, treatment of rosiglitazone was showed similar results with RGPM groups.

### Effect of RGPM on lipid and levels

The plasma triglycerides, total cholesterol, and LDL-cholesterol levels were significantly increased in HF diet group compared to control group. However, biochemical analysis of blood samples ofRGPM2 groups showed significant decrease of T-Cho and LDL-c when compared to HF diet group (Table 2), respectively (*P* < 0.05). Similarly, treatment of rosiglitazone group, T-Cho and LDL-c were significantly lower than those levels of HF diet group. Beside the plasma levels of HDL-c levels in RGPM 2 group increased compared to HF group (*P* < 0.05) (Table 2).

**Table 2:** Effect of RGPM on plasma lipid levels

Groups	Control	HF	HF		
			Ros.	RGPM 1	RGPM 2
T-Cho (mg/dl)	50.0 ± 2.0	8.5 ± 8.9**	51.8 ± 1.0^##^	66.3 ± 5.9	54.5 ± 39^#^
TG (mg/dl)	36.3 ± 5.8	240.8 ± 69.8*	107.8 ± 19.4	76.8 ± 16.6	181.8 ± 7.7
HDL-c (mg/dl)	24.0 ± 1.7	24.8 ± 0.8	31.5 ± l.2^##^	34.5 ± 2.9^#^	38.5 ± 3.8^#^
LDL-c (mg/dl)	14.5 ± 1.2	25.3 ± 3.0*	15.4 ± 3.5^#^	15.1 ± 2.8^#^	16.2 ± 1.4^#^

Values were expressed as mean ± S.E. (*n* = 10). *p < 0.05, **p < 0.01 vs. Cont.; ^#^ *p* < 0.05, ^##^ *p* < 0.01 vs HF. *Abbreviations*: *HF* high fructose, *HF + Ros.* high fructose diet with rosiglitazone, *HF + RGPM* high fructose diet with Red ginseng and *Polygoni Multiflori* Radix, *T-Cho* total cholesterol, *TG* triglyceride, *HDL-c* high-density lipoprotein cholesterol, *LDL-c* low-density lipoprotein cholesterol

Similar to the plasma lipid levels, the leptin and CRP levels were significantly increased in HF diet group compared to control group. However, treatment of RGPM groups, especially RGPM 2 group showed significantly decreased those levels compared to HF group (Table 3). Furthermore, although there was no significant difference of GOT levels in HF diet group compared to control group, treatment of RGPM 2 group showed significantly decreased the levels of GOT compared to HF group. Similarly, treatment of rosiglitazone was showed similar results with RGPM groups.

**Table 3:** Effect of RGPM on plasma parameters

Groups	Control	HF	HF		
			Ros.	RGPM 1	RGPM 2
GOT (IU/L)	9.7 ± 12.5	138.7 ± 4.7	54.5 ± 6.9^##^	100.5 ± 9.8	91.5 ± 1.9 ^##^
GPT (IU/L)	22.3 ± 4.5	30.0 ± 3.0	15.0 ± 0.9	25.3 ± 1.4	27.8 ± 1.3
Leptin (ng/ml)	2.42 ± 0.1	5.21 ± 0.2 **	2.85 ± 0.2^##^	2.98 ± 0.5^#^	3.51 ± 0.4^#^
CRP (mg/ml)	0.25 ± 0.01	0.30 ± 0.01**	0.24 ± 0.01^#^	0.22 ± 0.02^#^	0.15 ± 0.01^##^
Blood glucose (mg/dl)	143.3 ± 0.9	156.5 ± 34*	144.0 ± 3.1	148.8 ± 4.4	146.8 ± 1.5

Values were expressed as mean ± S.E. (*n* = 10). **p < 0.01 vs. Cont.; ^#^ *p* < 0.05, ^##^ *p* < 0.01 vs HF. *Abbreviations*: *HF* high fructose, *HF + Ros.* high fructose diet with rosiglitazone, *HF + RGPM* high fructose diet with Red ginseng and *Polygoni Multiflori* Radix, *GOT* glutamic-oxaloacetic transaminase, *GPT* glutamic-pyruvic transaminase, *CRP* C-reactive protein

### Effect of RGPM onbloodglucoselevel and oral glucose tolerance test

Non-fasting blood glucose levels were significantly increased in HF diet rats compared to control group at the 8 weeks. There was no significant change of blood glucose levels between HF group and rosiglitazone, RGPM groups (Table 3).

**Fig. 2:**
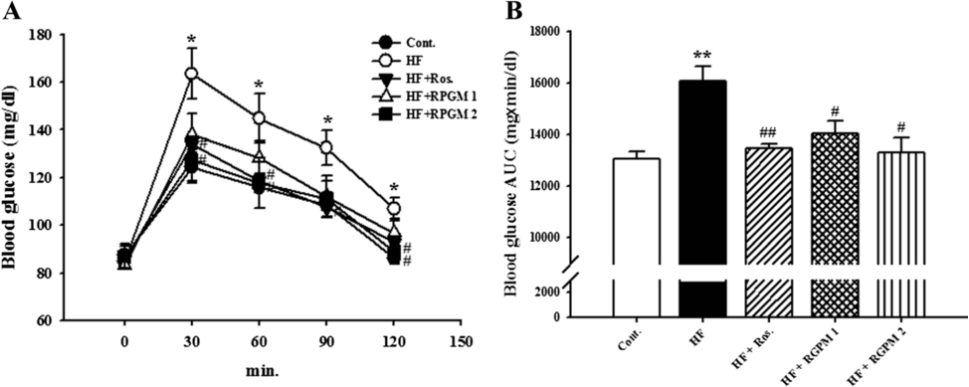
Effect of an RGPM on oral glucose tolerance test (**a**) and area under the glucose curve (**b**). Values were expressed as mean ± S.E. (*n* = 10). *p < 0.05, **p < 0.01 vs. Cont.; ^#^ *p* < 0.05, ^##^ *p* < 0.01 vs HF. *Abbreviations*: *HF* high fructose, *HF + Ros.* high fructose diet with rosiglitazone, *HF + RGPM* high fructose diet with Red ginseng and *PolygoniMultiflori* Radix

Oral glucose tolerance test was carried out to check insulin resistance in high-fructose diet rats after 8 weeks. The results showed that HF group maintained the significant increasein blood glucose levels at 30, 60, 90 and 120 min (*P* < 0.05), respectively. However, the plasma glucose levels in treatment of RGPM 2 group was significantly decreased at 30, 60 and 120 min as compared to HF group (*P* < 0.05) (Fig. 2a). Similarly, treatment of rosiglitazone group was significantly decreased at 30, 60, 90 and 120 min as compared to HF group. Moreover, area analysis (AUC) showed that HF diet group significantly increased compared to control group. However, treatment of rosiglitazone and RGPM groups were significantly decreased than that of HF group (Fig. 2b).

### Effect of RGPM on blood pressure

**Fig. 3:**
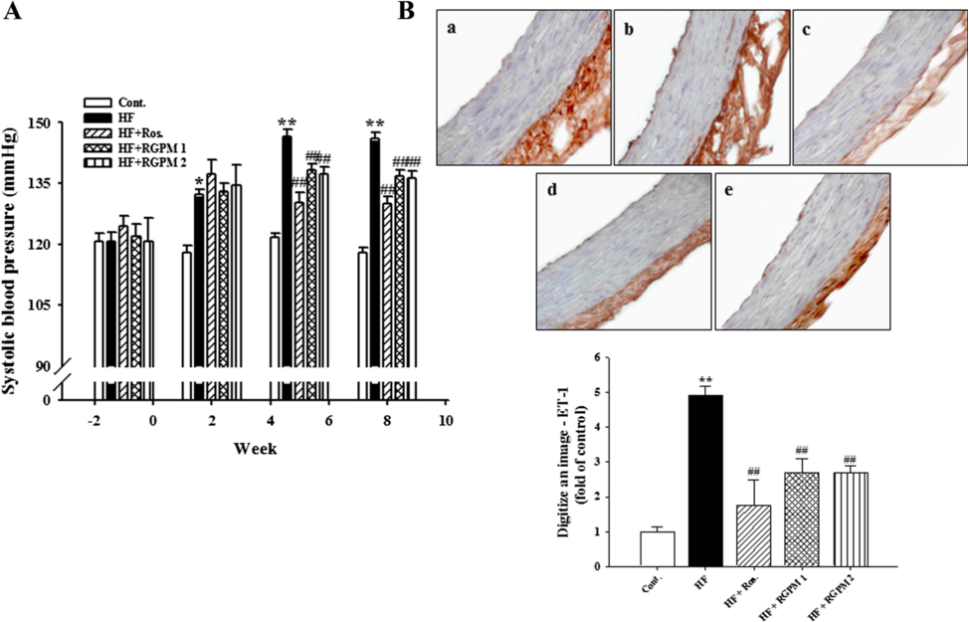
Effects of RGPM on systolic blood pressure (**a**) and expression of ET-1 (**b**) immunereactivity in aortic tissues of HF diet rats. Representative immunohistochemistry (magnification × 400) and quantifications are shown. Values were expressed as mean ± S.E. (*n* = 10 at (**a**), *n* = 4 at (**b**)). **p < 0.01 vs Cont.; ^#^ *p* < 0.05, ^##^ *p* < 0.01 vs HF

At the beginning of the experimental feeding period, the levels of systolic blood pressurein all groups were approximately 115 ~ 125 mmHg as investigated by the tail-cuff technique. After 8 weeks, systolic blood pressure of HF group was significantly increased than that of control group (*P* < 0.01). However, treatment of RGPM groupswassignificantly decreased than that of HF group during all experimental period (*P* < 0.01) (Fig. 3a). Similarly, treatment of rosiglitazone was showed similar results with RGPM groups. In addition, the protein expression of ET-1 level was increased in the HF diet group compared to control group. However, treatment of RGPM groups was significantly decreased expression levels of protein compared to HF group (Fig. 3b).

### Effect of RGPM on the expressions levels of adhension molecules, PPAR-γ and ET-1 in aorta

**Fig. 4:**
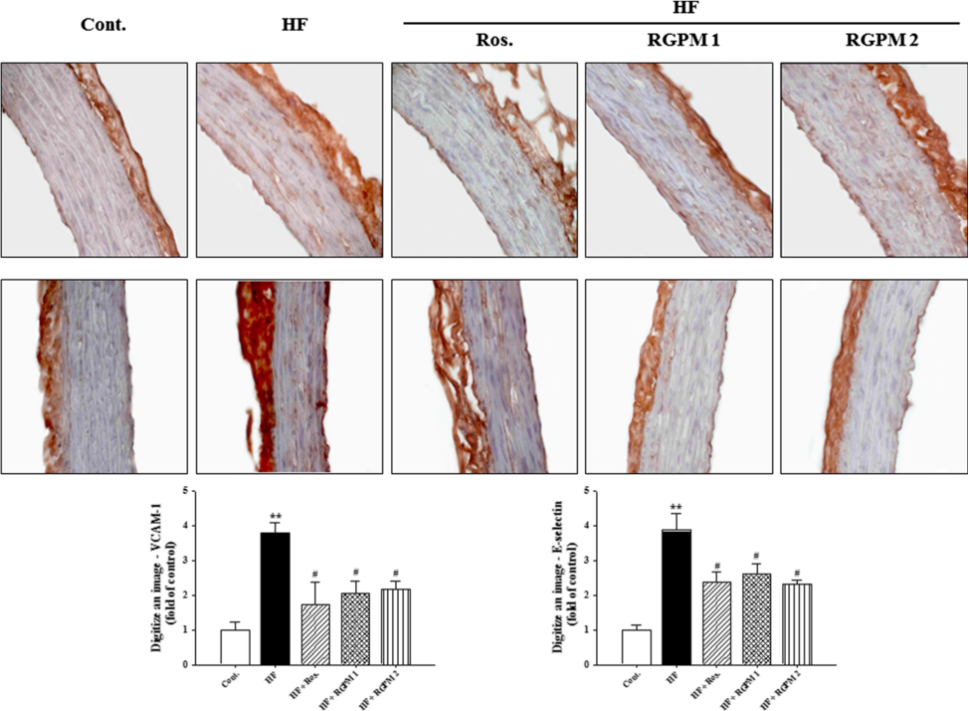
Effects of RGPM on VCAM-1 (*upper*) and E-selectin (*middle*) immunoreactivity in aortic tissues of HF diet rats. Representative figure (magnification × 400) and quantifications are shown (*lower*). Values were expressed as mean ± S.E. (*n* = 4). **p < 0.01 vs Cont.; ^#^ *p* < 0.05, ^##^ *p* < 0.01 vs HF

Immunohistochemistry was performed to determine the direct expression of adhension molecules in the aortic wall. Adhension molecules expression such as VCAM-1 and E-selectin were increased in the HF diet group (*P* < 0.01). However, treatment of RGPM groups was significantly decreased expression levels of protein (VCAM-1 and E-selectin, *P* < 0.05) (Fig. 4). Similarly, treatment of rosiglitazone was showed similar results with RGPM groups.

**Fig. 5:**
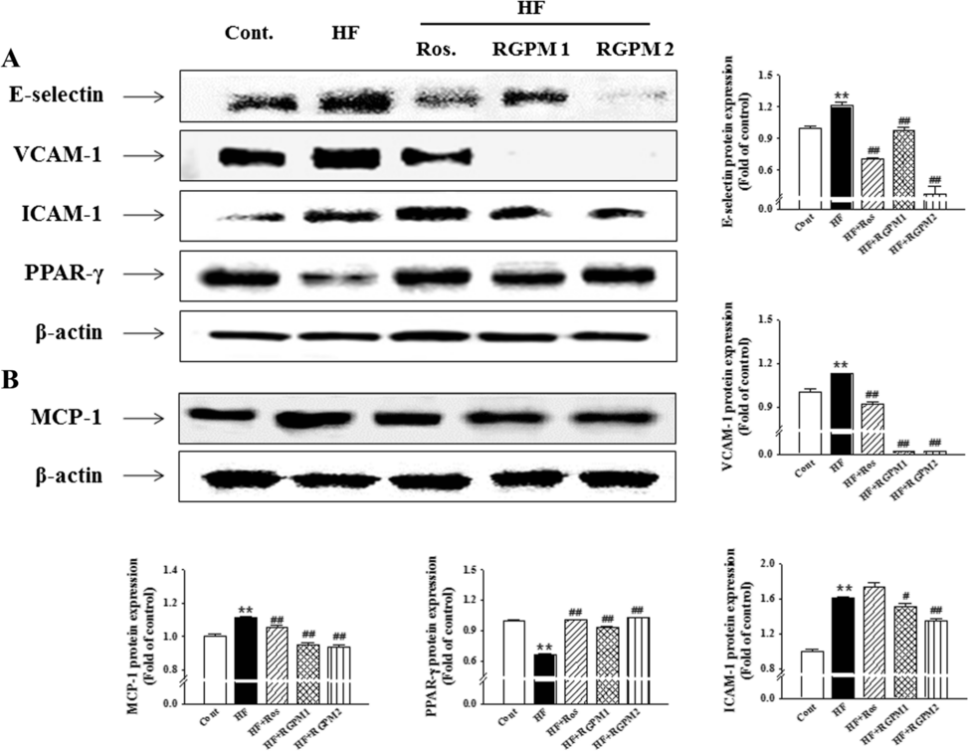
Effect of RGPM on the expression of adhension molecules and PPAR-γ in the aorta (**a**) and MCP-1 in the liver (**b**) of HF diet rats. Each electrophoretogram is representative of the results from three individual experiments. Values were expressed as mean ± S.E. (*n* = 4). **p < 0.01 vs Cont.; ^#^ *p* < 0.05, ^##^ *p* < 0.01 vs HF

The protein expression of VCAM-1, ICAM-1, E-selectinand PPAR-γ in the descending aortas of all groups of rats was examined by Western blot analysis. Expression of adhension molecules (VCAM-1, ICAM-1 and E-selectin) levels were increased in the HF diet group compared to control group. However, treatment of RGPM groups was significantly decreased expression levels of protein compared to HF group (Fig. 5). Moreover, we examined the expression of PPAR-γ levels to evaluate vascular endothelial function and inflammation. The PPAR-γ protein levels decreased in the HF diet group compared to control group. However, RGPM groups were recovered expression levels of protein compared to HF group (Fig. 4). Similarly, treatment of rosiglitazone was showed similar results with RGPM groups.

### Effect of RGPM on the hepatic lipids

**Fig. 6:**
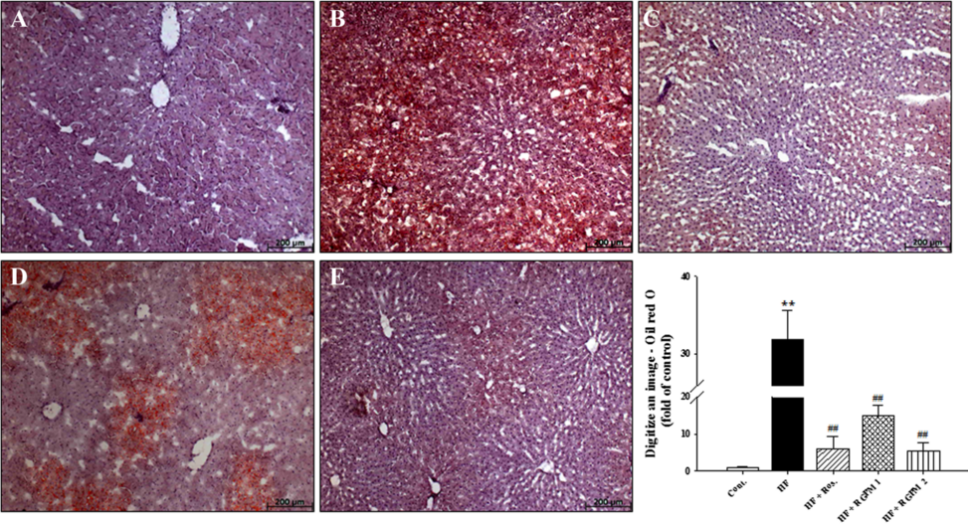
Representative microscopic photographs of Oil red O stained section of the liver in HF diet rat. Representative Oil red O staining (magnification × 100) and quantifications are shown. Values were expressed as mean ± S.E. (*n* = 4). **p < 0.01 vs Cont.; ^##^ *p* < 0.01 vs HF

To investigate the existence of fat accumulation of liver in all experimental groups, we prepared frozen section of liver and stained with Oil red O. Lipid droplets were detected in HF diet groups. However, treatment of RGPM groups showed the number of lipid droplets significantly decreased compared to HF diet group (Fig. 6). Especially, treatment of RGPM 2 was more effectively suppressed lipid accumulation. Treatment of rosiglitazone was showed similar results with RGPM groups.

## Discussion

Herb, Acupuncture and Natural Medicine (HAN), one of the most ancient and revered forms of healing, has been used to diagnose, treat, and prevent disease for over 3000 years [22]. HAN is now used worldwide as an effective means of overcoming disease. Red ginseng and *Polygoni Multiflori* Radix, are well-known widely used traditional medicinal herb specifically for promoting blood circulation to maintain body homeostasis [21].

Fructose is a lipogenic component, its consumption promotes the development of atherogenic lipid profile and elevation of postprandial hypertriglycemia [23, 24]. In addition, HF diet animals develop hypertriglyceridemia, obesity, impaired glucose tolerance, fatty liver, increased SBP and vascular remodeling [25, 26]. Here we provided the first evidence for the beneficial effect of the combination with Red ginseng and *Polygoni Multiflori* Radix on metabolic disordersin high fructose-induced metabolic syndrome rat model. Several studies already reported Red ginseng has beneficial effects against obesity, hyperglycemia [27, 28]. Moreover, red ginseng also has beneficial effective anti-inflammatory therapeutic for the atherosclerosis [29]. In addition, some studies already reported *Polygoni Multiflori* Radix regulates lipid homeostasis and fat metabolism [30, 31]. In this case, we hypothesized that the combination with Red ginseng and *Polygoni Multiflori* Radix shows a higher synergies effect of prevention of metabolic syndrome, such as hypertension, obesity and hyperlipidemia et al.

The development of leptin resistance classically has been characterized by increased body fat and elevated circulating leptin levels [32]. In present study, HF diet increased of leptin level, and induced development of leptin resistance. Also, HF diet increased body weight and adipocyte size. However, treatment RGPM improved leptin resistance with the amelioration of down regulation of body weight and adipocyte size. These results suggest that RGPM may be useful to suppress the development of leptin resistance leading to obesity. Recently there is a strong correlation between low activation state of AMPK with metabolic disorder associated with insulin resistance, fat deposition and dyslipidemia [33, 34]. It is suggested that fructose-driven leptin resistance in the present study maybe associated with impaired leptin-mediated decrease in AMPK phospholyation. Although, we did not examine specific research related energy metabolism as AMPK pathway, we conjectured treatment of RGPM would be related to several improvements of metabolic syndrome by activating of AMPK related signal pathways.

NFALD where triglyceride is stored more than 5 % in the liver is a status in which fat is accumulated in the whole liver tissues by insulin resistance, obesity and dyslipidemia [35]. It is known that overallmetabolic disorderinduces increasing ofoxidative stress, increasing inflammatory cytokine such as PAI-1 and MCP-1. Also, it is decreasing secretion of adiponectin which functions reversely is a cause of atherosclerosis outbreak [36]. In addition, Dyslipidemia, impaired glucose tolerance and fatty liver are major features associated with metabolic syndrome in HF diet rats [37, 38]. Fructose induces impaired glucose tolerance via the elevation of plasma triglyceride levels. In addition, previous study demonstrated that an elevated fructose diet associated with impaired glucose tolerance and endothelial dysfunction precedes the development of hypertension [39]. Impaired glucose tolerance plays an important role in the development of such abnormalities as insulin resistance, type 2 diabetes and dyslipidemia [40]. HF diet induced impaired glucose tolerance, dyslipidemia and fatty liver, whereas, treatment RGPM improved impaired glucose tolerance with the amelioration of dyslipidemia and fatty liver. Thus, these results suggest that RGPM may be useful to suppress the development of atherosclerotic lesions and ameliorated lipid metabolism in metabolic syndrome model. Although, in the present study did not confirm a secretion of factor of PAI-1 or adiponectin, it is well known that CRP is a factor of direct inducer of PAI-1. This study showed that RGPM decreased of levels of CRP as well as reduction of weight and size of fat tissues and increased of leptin sensitivity. In this way, these results suggest that RGPM that changed fatty metabolism for has effects to secretion of PAI-1 or adiponectin. Besides, treatment of RGPM suppressed expression of MCP-1 which is improved of by NFALD induced steatohepatitis.

It is reported that the inflammation index of interior of body is a predictive progressivefactor of cardiovascular disease caused by atherosclerosis, metabolic syndrome and type 2 diabetes. High-sensitivity CRPis well known as the most powerful predictive factor of cardiovascular disease, which caused an increase of inflammatory cytokine, such as MCP-1. CRP promotes a secretion of triglyceride and it highly came out from patients of cardiovascular disease [41]. It is reported that high-sensitivity CRP induced high blood pressure and progress of blood vessel inflammation, which is related with adhesion molecules, ET-1 and inflammatory cytokine in vascular endothelial of a blood vessel and increased in an initial stage of atherosclerosis [42, 43]. In the present study, RGPM ameliorated vascular inflammation by down-regulation of ET-1 as well as ICAM-1, VCAM-1 and E-selectin expression in thoracic aorta. Moreover, RGPM suppressed the levels of CRP in plasma and down-regulated blood pressure, compatible with the processes of atherosclerosis. Therefore, improvement of endothelial function is predicted to regulate lipid homeostasis [44]. Thus, anti-hypertension and anti-vascular inflammatory effects of RGPM contribute to the beneficial effects on endothelial function and lipid metabolism in metabolic syndrome.

In the present study used rosiglitazone, selective hypoglycemic agent, as a positive control, which is recently reported that rosiglitazone effect reducing of fat accumulation, blood pressure and improve vascular inflammation in NFALD [45, 46]. In addition, clinical trial showed that glitazone medicine improved insulin resistance of NASH patients, morphological change and stabilized an index of GPT and GOT [47]. To investigate compare of damage of liver with aggravation of inflammation, this study measured plasma levels of GPT and GOT. Treatment of RGPM significantly improved the levels of GOT. Even though the levels of GPT did not showed significant difference, judging from the decrease results speculated that RGPM has effect of an improvement by high fructose induced liver damage.

## Conclusions

These results suggest that RGPM ameliorates lipid metabolism, impaired glucose tolerance, hypertension, fatty liver and obesity in HF diet-induced metabolic syndrome, at least in part, via reducing of inflammation. Therefore, combination of Red ginseng and Polygoni Multiflori Radix might be a beneficial therapeutic approach for metabolic syndrome.

## Abbreviations

CRP: C-reactive protein
ET-1: Endothelin-1
GOT: Glutamic-oxaloacetic transaminase
GPT: Glutamic-pyruvic transaminase
HDL: High-density lipoproteincholesterol
HF: High fructose
ICAM-1: Intercellular adhesion molecule 1
LDL: Low-density lipoproteincholesterol
MCP-1: Monocyte chemoattractant protein-1
OGTT: Oral glucose tolerance test
PPAR-γ: Peroxisome proliferator-activated receptor gamma
RGPM: Red ginseng and *Polygoni Multiflori* Radix
TG: Triglyceride
VCAM-1: Vascular cell adhesion molecule-1
